# Electrical responses of three classes of granule cells of the olfactory bulb to synaptic inputs in different dendritic locations

**DOI:** 10.3389/fncom.2014.00128

**Published:** 2014-10-13

**Authors:** Fábio M. Simões-de-Souza, Gabriela Antunes, Antonio C. Roque

**Affiliations:** ^1^Laboratory of Neural Systems (SisNE), Department of Psychology, Faculdade de Filosofia Ciencias e Letras de Ribeirão Preto, Universidade de São PauloRibeirão Preto, Brazil; ^2^Center for Mathematics, Computation and Cognition, Federal University of ABCSão Bernardo do Campo, Brazil; ^3^Laboratory of Neural Systems (SisNE), Department of Physics, Faculdade de Filosofia Ciencias e Letras de Ribeirão Preto, Universidade de São PauloRibeirão Preto, Brazil

**Keywords:** granule cells, olfactory bulb, active dendrites, spikes, dendrodendritic synapses

## Abstract

This work consists of a computational study of the electrical responses of three classes of granule cells of the olfactory bulb to synaptic activation in different dendritic locations. The constructed models were based on morphologically detailed compartmental reconstructions of three granule cell classes of the olfactory bulb with active dendrites described by Bhalla and Bower (1993, pp. 1948–1965) and dendritic spine distributions described by Woolf et al. (1991, pp. 1837–1854). The computational studies with the model neurons showed that different quantities of spines have to be activated in each dendritic region to induce an action potential, which always was originated in the active terminal dendrites, independently of the location of the stimuli, and the morphology of the dendritic tree. These model predictions might have important computational implications in the context of olfactory bulb circuits.

## Introduction

Computational models of the olfactory system have produced several important contributions about the functioning of this system, which has been considered a model system for computational neuroscience (Davis and Eichenbaum, 1991; Cleland and Linster, 2005; Simões-de-Souza and Antunes, 2007). But, despite the large number of computational models of olfactory bulb networks using neurons with simplified dendritic trees of granule cells (Davison et al., 2003; Simões de Souza and Roque, 2004; Yu et al., 2013; Kaplan and Lansner, 2014; Migliore et al., 2014), little has been done to investigate the outcome of the complex morphology of the different classes of granule cells of the olfactory bulb with active dendrites in the integration of their synaptic inputs.

Granule cells are the most numerous inhibitory interneurons present in the olfactory bulb, which has a proportion of 100–200 granule cells to each mitral and tufted cell (Saghatelyan et al., 2003; Shepherd et al., 2007). New granule cells develop constantly in the olfactory bulb through a process of neurogenesis and cell migration that is implied with synaptic plasticity and memory (Nissant et al., 2009; Sakamoto et al., 2014). In the olfactory bulb, granule cells play a key role in the information processing of the olfactory system (Shepherd et al., 2007; Labarrera et al., 2013).

The terminal dendrites of the granule cells have active membrane properties that can boost the excitability of the neurons to excitatory synaptic inputs impinging in different dendritic locations (Pinato and Midtgaard, 2005; Balu et al., 2007). These terminal dendrites make dendrodendritic synapses with the lateral dendrites of the mitral and tufted cells, which are the principal neurons of the olfactory bulb. Glutamate is released from the dendrites of the mitral/tufted cells that excite glutamatergic receptors in the spines of the granule cells. Presumably, the stimulation of AMPA, and NMDA type synapses lead to calcium inflow mainly through NMDA receptor channels in the spines of the granule cells (Schoppa et al., 1998), which induces the release of GABA in the synaptic cleft promoting the inhibition of the secondary dendrites of the mitral/tufted cells (Rall et al., 1966; Rall and Shepherd, 1968). Thus, the spines of the granule cells produce dendrodendritic recurrent inhibition of the mitral/tufted cells and lateral inhibition between mitral/tufted cells (Rall et al., 1966; Yokoi et al., 1995). Moreover, the dendritic trunk and deep dendrites of the granule cells can receive centrifugal fiber (CF) inputs from several brain regions that modulate the activity of the olfactory bulb (Laaris et al., 2007; Whitman and Greer, 2007; O'Connor and Jacob, 2008; Doucette et al., 2011).

Accordingly with morphological criteria there are three classes of granule cells in the olfactory bulb termed type I, II, and III (Mori et al., 1983; Woolf et al., 1991; Shepherd et al., 2007). The type I granule cell has terminal dendrites that branches and has spines present throughout the external plexiform layer. The type II granule neuron has branching patterns that are confined to the lower one-half to one-third of the external plexiform layer. The type III granule cell arbors extensively only in the upper one-half to one-third of the external plexiform layer. Despite the morphological differences among different classes of granule cells that have been long recognized, it remains unclear whether the presence of active dendrites in each one of them induce different electrical responses to the same pattern of synaptic inputs. To investigate this aspect, we developed a computational model of three classes of granule cells with distinct distributions of dendritic spines along the active dendrites to investigate how each class integrate their its synaptic inputs.

## Materials and methods

The computational models used to simulate the granule neurons were based on Bhalla and Bower detailed compartmental reconstructions of the three granule neuron classes of the olfactory bulb with active properties (Bhalla and Bower, 1993). Bhalla and Bower models are the most detailed reconstructed compartmental models with conductance based active dendrites available in the literature, and the reader is referred to the original paper to obtain more information about their development (Bhalla and Bower, 1993). The original model neurons were modified to include distinct distributions of dendritic spines (Woolf et al., 1991) that receive glutamatergic excitatory synapses on their dendritic spines containing NMDA and AMPA receptors.

The source code of the computational model is available at: https://senselab.med.yale.edu/modeldb/ShowModel.asp?model=156828, password=1416.

### Granule cells

To investigate the effect of active dendrites on the integration of synaptic inputs in the three classes of granule cells, we kept the original compartmental composition and number of active channels of the type I, II, and II granule cells unchanged (Bhalla and Bower, 1993), which include the rat brain sodium, potassium, anomalous rectifier potassium and non-inactivating muscarinic potassium currents (Bhalla and Bower, 1993). Then, we modified the distributions of the spines in the terminal dendrites, dendritic trunk, and deep dendrites of the model cells to reproduce the detailed location, number and type of spines of a representative granule neuron of each class reconstructed in camera lucida (Woolf et al., 1991).

**Figure 1:**
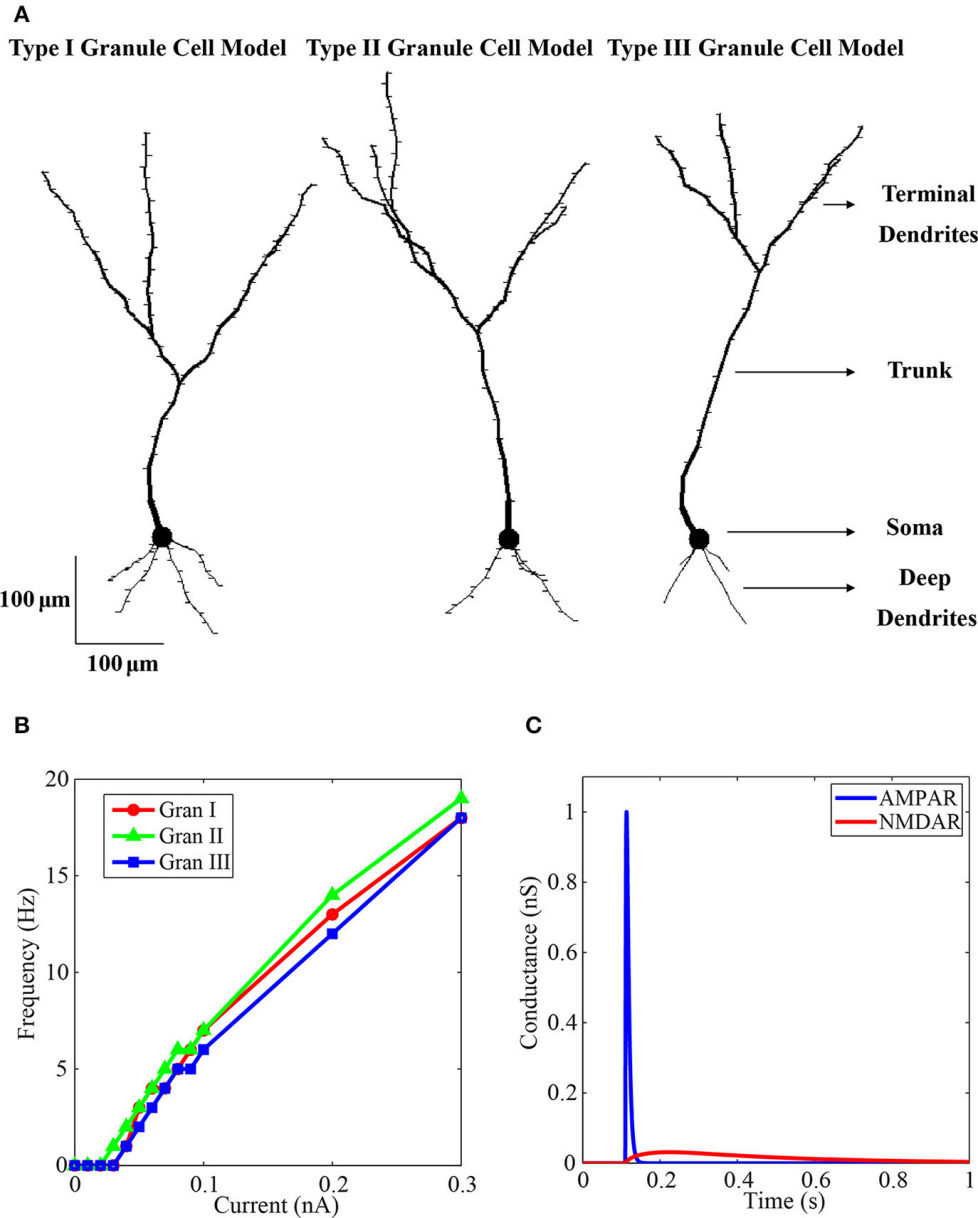
**Granule neuron model. (A)** Morphology of the final model neurons with the dendritic spines. At left is the type I granule neuron model with 112 compartments and 112 spines, in the middle is the type II granule neuron with 114 compartments and 114 spines, and at right is the type III granule neuron with 89 compartments and 61 spines. Each model neuron has a soma, a trunk, the deep dendrites and the terminal dendrites. **(B)** Curves of injected current in the soma vs. spike frequency of the three simulated granule neurons. The markers are the simulated points. There are more points to the weak currents to determine the spike threshold of the neurons. The red line shows the firing rate of the type I granule neuron, the green line shows the firing rate of the type II granule neuron, and the blue line shows the firing rate of the type III granule neuron. **(C)** Activation of the model AMPA (blue line) and NMDA (red line) receptor channel conductances by glutamate. The concentration of ion magnesium was 1.2 mM.

There are 194 pedunculated spines in the type I experimentally reconstruct granule neuron, 118 pedunculated spines in the type II granule neuron, and 114 spines in the type III granule neuron. The type I and II have more pedunculated spines near the soma than the type III (Woolf et al., 1991). Each spine was simulated as two compartments representing the neck and the head. We included one spine per granule cell compartment. Because the number of compartments was smaller than the number of peduculated spines observed experimentally, the membrane area of these missing spines was taken in consideration by increasing the membrane area of the cell with the area of the missing spines, considered as 3.37 μm^2^ per missing spine. According to morphological data, the type III granule cell was simulated with no spines in the proximal trunk and deep dendrite (Woolf et al., 1991). The final constructed model for the type I granule neuron has 112 compartments and 112 simulated spines, the type II granule cell has 114 compartments and 114 simulated spines, the type III granule cell has 89 compartments and 61 simulated spines (Figure 1A, Supplementary Table 1).

The curves of spike frequency per injected current in the soma of the three simulated granule cells are shown in Figure 1B, which is a standard procedure to characterize computational model of single cells. The curves were very similar in the three model cells, in particular the firing threshold of the type I and II model cells is 0.04 nA, and the threshold of the type II is 0.03 nA.

A resting membrane potential of −65 mV was adopted for all granule cells, which is inside a range of values obtained with *in vivo* whole-cell recording in the rat olfactory bulb (Cang and Isaacson, 2003).

### Dendritic spines

Each simulated spine has two cylindrical compartments, one representing the spine neck with a diameter of 0.23 μm and length 1.9 μm and another representing the spine head with a diameter of 0.8 μm and a length of 0.8 μm (Woolf et al., 1991). The spines were simulated containning both AMPA and NMDA receptor channels and a thin shell model immediatelly beneath the membrane that receive the calcium that flows through the activated NMDAR channels. The parameters used to simulate of these channels were based mainly in the Davison's model (Davison et al., 2003), which were obtained from the experimental works of Schoppa et al. (1998).

The AMPAR channel was simulated according to Equation (1):

(1) GAMPAR(V, t)=gAMPAR(e(−tτ1)−e(−tτ2))τ1−τ2

where *G*_*AMPAR*_ is the calculated AMPA conductance, *g*_*AMPAR*_ = 1 nS is the maximal conductance of the channel, and τ_1_ = 2 ms, and τ_2_ = 5.5 ms are the rising and decaying time constants, respectively (Davison et al., 2003).

The NMDA receptor model was based on Equation (2) (Zador et al., 1990):

(2) GNMDAR(V, t)=gNMDAR(e−tτ1−e−tτ21+η[Mg2+]e−γV)

which considers the voltage-dependent blocking of the channel by the ion magnesium. *G*_*NMDAR*_ is the calculated NMDAR conductance, *g*_*NMDAR*_ = 0.593 nS is the maximal conductance of the channel, τ_1_ = 52 ms and τ_2_ = 343 ms are the rising and decaying time constants, respectively (Davison et al., 2003), [Mg^2+^] = 1.2 mM is the ion magnesium concentration, η = 0.2801 and γ = 62.

The current through the channels was calculated by Equation (3),

(3) $$I(t)=G(t)\left(E_{K}-V_{m}\right)$$

where *I* is the calculated current, *G* is the calculated channel conductance (*G*_*NMDAR*_ or *G*_*AMPAR*_), *V*_*m*_ is the compartmental membrane potential and *E*_*K*_ is the reversal potential of the channel, considered zero for the AMPA receptor channels.

An example of the time variations of the AMPA and NMDA receptor channel conductances of the model in response to glutamate activation is shown in Figure 1C. The maximum AMPAR conductance is 1 nS. There is a fast rising and fast decaying time in the variation of the AMPAR conductance (Figure 1C, blue) when compared with the NMDAR conductance that has very slow rising and decaying time constants (Figure 1C, red). Because of the blocking of the NMDAR by 1.2 mM of ion magnesium the maximum conductance of this channel, which is 0.593 nS, was not reached. The shell used to simulate the calcium dynamics was based on Equation (4) (Traub et al., 1991; Bower and Beeman, 2007):

(4) $$\frac{d[Ca^{2+}]}{dt}=BI_{Ca}-r{\text{Ca}}^{2+}$$

where the parameters were calculated for the spine dimensions considered here. *B* = 5.2 · 10^−6^/(*a* · *L*) is the ion calcium diffusion rate constant of the shell, where the spine head area is *a* = 2 μm^2^ and the shell thickness is *L* = 0.1 μm. Because of buffering factors *B* = *B*/10, then *B* = 26 · 10^11^. [Ca^2+^] is the calculated ion calcium concentration on the shell, *I*_*Ca*_ is the inward ion calcium current coming from NMDAR channel, *r* = 870 s^−1^ is the extrusion rate of ion calcium (Egger and Stroh, 2009). The resting intracellular ion calcium is 0.05 μM (Egger and Stroh, 2009).

The fractional calcium concentration (*P*_*f*_) through NMDAR channels followed the Equation (5) (Schneggenburger, 1996):

(5) $$P_{f}=\frac{{\left[Ca^{2+}\right]}_{\text{o}}}{{\left[Ca^{2+}\right]}_{\text{o}}+{\left(\frac{PM}{PCa^{2+}}\right)}^{-1}\frac{\left[M\right]}{4}\left(1-e^{\left(2V\frac{F}{RT}\right)}\right)}$$

where [Ca^2+^]_o_ is the extracellular free Ca^2+^ concentration (2 mM), V is the membrane potential in the spine head, F is the Faraday constant (96,485 C·mol^−1^), *R* is the gas constant (8.314 J·K^−1^·mol^−1^), *T* is the temperature in degree Kelvin (298.15 K), *M* is the monovalent ion concentration (155 mM), and PCa^2+^/PM is the permeability ratio of Ca^2+^ over monovalent ions (3.6). *P*_*f*_ = 15% at the membrane resting potential.

The reversal potential (*V*_*r*_ = 2.18 mV) for the NMDAR channel was obtained from the extended Goldman-Hodgkin-Katz (GHK) Equation (6) (Jan and Jan, 1976; Schneggenburger, 1996):

(6) Vr=RTFln(4[M]([M]+4PCa2+PM[Ca2+]o))122[M]

### Simulations

The simulations were performed on the GENESIS simulator (Bower and Beeman, 2007). We utilized the Crank–Nicolson implicit numerical method to solve the differential equations and all the simulated data were saved in files and processed using the MATLAB package (Mathworks Inc.)

Three series of simulations were performed with the granule cell models. The first series of simulations was used to test the model of dendritic spines implemented with its synaptic channels and calcium shell. The second series of simulations was used to investigate the effect of synaptic activation in different locations of the dendritic tree of each simulated cell on the generation of excitatory post synaptic potentials (EPSPs) and action potentials in the soma. The third series of simulations was performed to determine the origin of the generation of action potentials by simultaneous measurements in the terminal location of the dendritic trunk and in the soma during synaptic stimulation in different positions of the dendritic tree.

## Results

### Dendritic spine model

**Figure 2:**
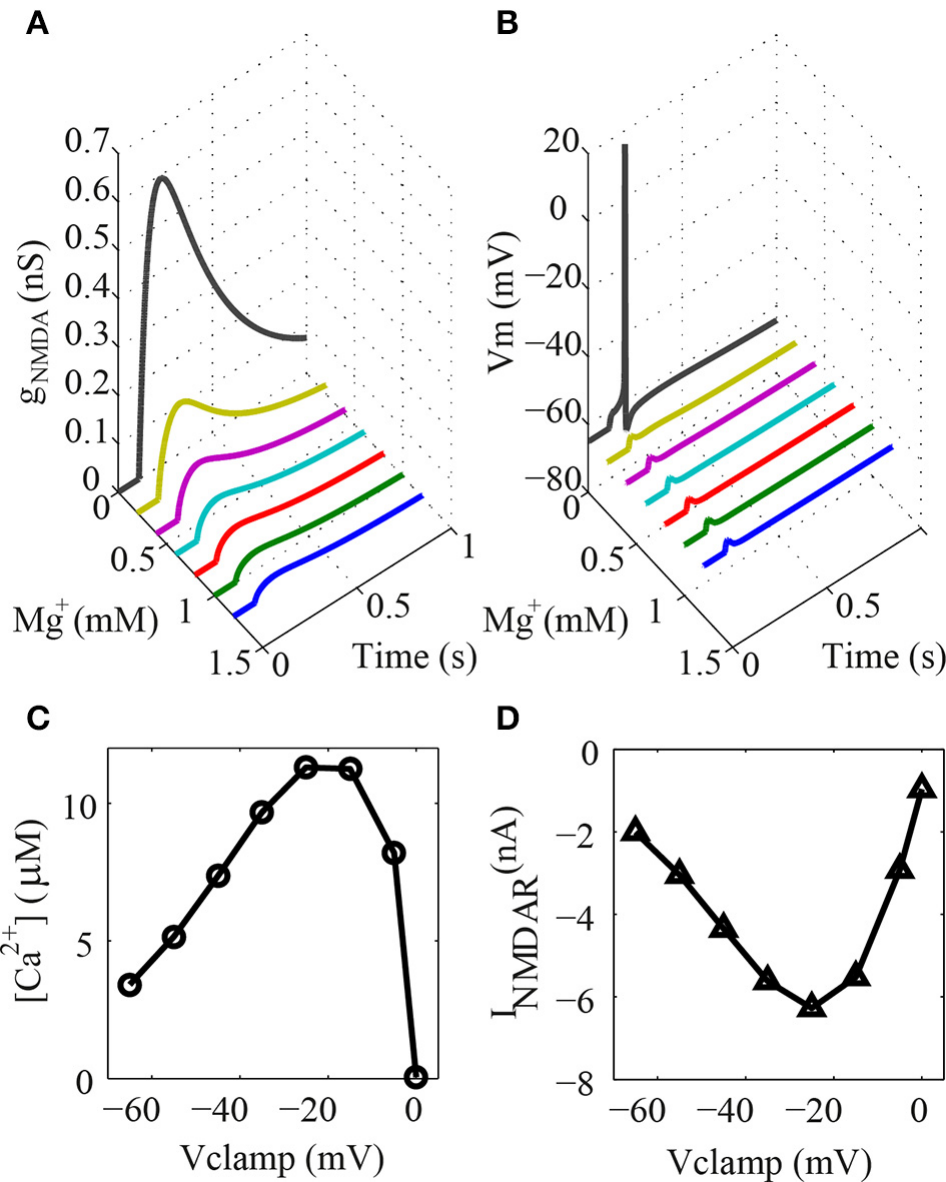
**Dendritic spine model. (A)** NMDA conductance on the dendritic spine with varying concentrations of ion magnesium during a synaptic activation. Note that the reference level of ion magnesium is 1.2 mM **(B)** Somatic EPSP to the same situation described in **(A)**. **(C)** Concentration of ion calcium in the spine shell voltage-clamped at different potentials during synaptic activation. **(D)** Voltage dependence of the NMDAR current to the same situation described in **(C)**.

The electrical response of the type I granule neuron to a glutamatergic activation of only one spine at the tip of a terminal dendrite to different values of [Mg^2+^] was simulated to test the dendritic spine model (Figure 2A). The results obtained demonstrate that the conductance of the channels increase gradually with the reduction of the [Mg^2+^]. The somatic EPSP during different [Mg^2+^] is shown in Figure 2B. The head of the spine was voltage clamped at different levels to verify the intracellular concentration of calcium ions resulting from the NMDAR currents (Figures 2C,D), This is a typical curve for the voltage dependence of the NMDAR current (Figure 2D) and the voltage dependence of the Ca^2+^ flux through NMDAR channels (Figure 2C) (Garaschuk et al., 1996).

The simulation of the dendritic spine model showed that both the NMDAR and AMPAR conductances and the influx of ion calcium into the spine head shell is working as expected after synaptic activation (Supplementary Figure 1). In particular, the model simulated the crucial role of the ion magnesium blocking on the NMDA receptors. Since the ion calcium influx in the spines mediates the GABA release of the reciprocal synapses of the olfactory bulb (Chen et al., 2000), the present model can be used to simulate the dendrodendritic interactions between the granule neurons and mitral/tufted cells.

### Effects of the synaptic inputs in different locations of the dendritic tree

**Figure 3:**
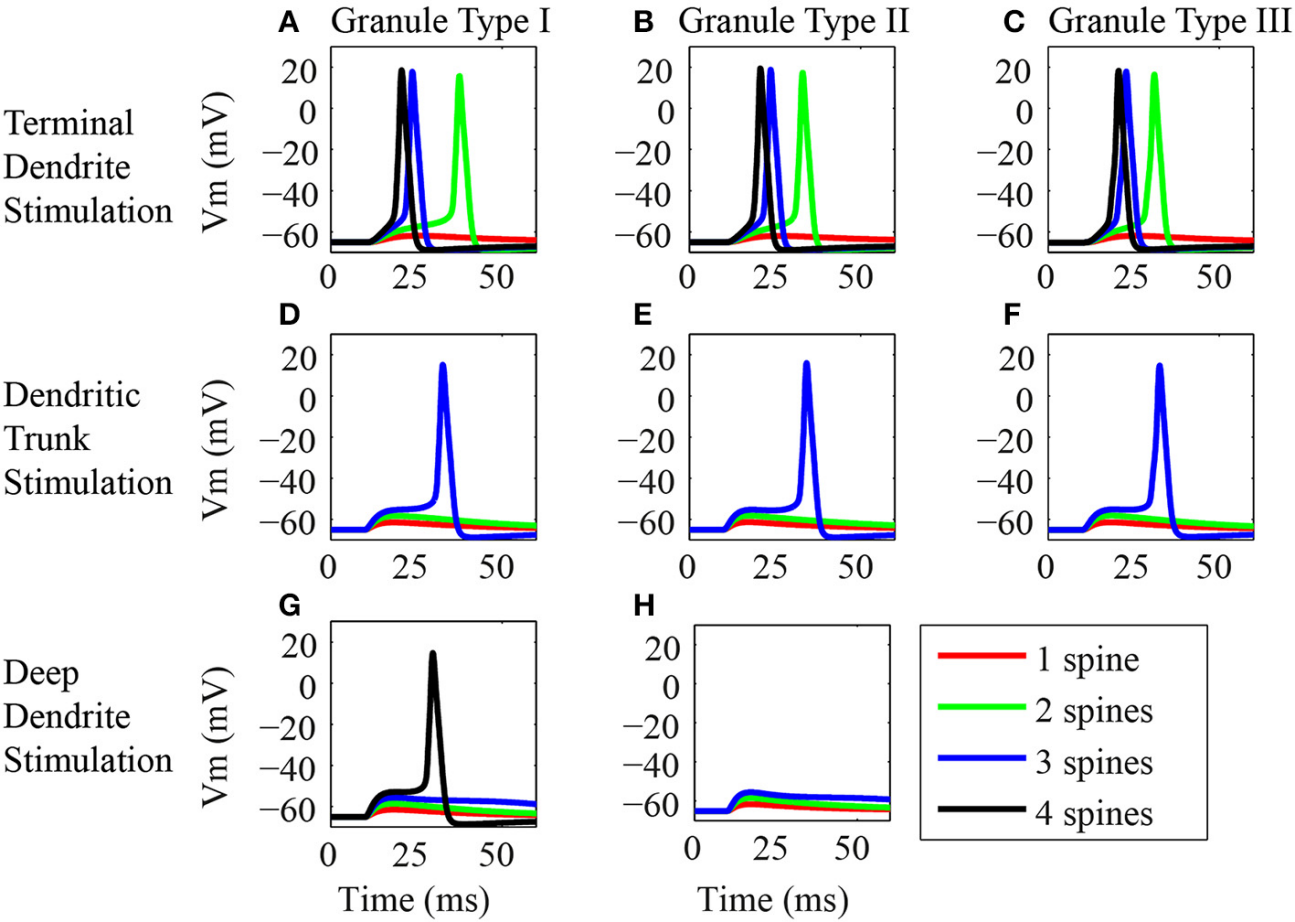
**Synaptic inputs in different dendritic locations. (A–H)** Somatic EPSP or action potential responses of the three classes of granule neuron models to synaptic activation in different locations of the dendritic tree. In the left column **(A,D,G)** are the panels showing the responses of the type I granule neuron model, in the middle **(B,E,H)** are the responses of the type II granule neuron model, and in the right **(C,F)** are the responses of the type III granule neuron model. The top row **(A–C)** shows the responses to stimulations of the spines located in the terminal dendrite. The middle row **(D–F)** shows the responses to stimulations of the spines located in the trunk. The bottom row **(G,H)** shows the responses to stimulations of the dendrites located in the deep dendrite. Only the type III granule neuron models have no spines in the deep dendrites. Note that several spines were stimulated in each location, where red, green, blue, and black traces are respectively, the responses of the granule neuron models to stimulation of one, two, three, and four dendritic spines. The stimulus consists of the synaptic activation of NMDA and AMPA receptors by glutamate in the dendritic spines. The black line is absent in some panels because the stimulation of up to three spines in the dendritic trunk was sufficient to generate an action potential. In addition, type II granule cells have only three deep dendrites and therefore only three spines were stimulated in the deep dendrites of these cells.

The results of the study of the impact of the synaptic activation in different locations of the dendritic tree in the generation of EPSPs and action potentials for three classes of granule cells are presented in Figure 3. The stimuli consist in the synaptic activation of NMDA and AMPA receptors in the spines located at the tip of the terminal dendrites, in the dendritic trunk or in the tip of the deep dendrites. We varied the number of activated spines for each of these three locations. We stimulated spines from different dendritic branches but located in the same horizontal plane of the terminal and deep dendrites. In the trunk, we increased the number of activated spines from the nearest spine to the soma to the next.

The responses of the three classes of granule cells to the stimulation of the spines located in the terminal dendrites were very similar (Figures 3A–C). The stimulation of only one spine in this location produced a slight depolarization of the postsynaptic membrane (red trace), however, the stimulation of more than one spine induced action potentials in all three cells (green, blue and black traces). The higher the number of activated spines, the lower was the latency for the occurrence of the spikes.

The electrical responses of the three classes of granule cells to stimulation of the spines on the dendritic trunk were again very similar (Figures 3D–F). The stimulation of only one or two spines produced a small depolarization of the membrane potential (red and green traces), however, the stimulation of three spines induced action potentials in all three cells (blue traces). The amplitude of the EPSP increased with the number of activated spines (read and green traces). Although the type III granule cell has no spines on proximal dendritic trunk, the stimulation of the spines located in the distal dendritic trunk produced similar responses to the ones obtained by stimulating the proximal spines of the type I and II granule cells (Figures 3D–F, blue traces).

The types I and II granule cells show equivalent electrical responses to stimulation of the spines in the deep dendrites (Figures 3G,H). Since the type III granule cell model has no spines in the deep dendrites, it was not stimulated. The stimulation of one to three spines in this location produced only an EPSP in the types I and II granule cells. The EPSP response increases with the number of activated spines (red, green, and blue traces). The stimulation of four spines in the type I granule cell induced an action potential (black trace). Note that the type I granule cell had four branches of deep dendrites and the type II granule cell had only three branches of deep dendrites (Figure 1A).

The study of the effect of the synaptic activation in different positions of the dendritic arbor of the three classes of granule cells did not find strong differences between the cells. However, it showed significant differences in the response of each type of granule cell to the activation of the spines in different locations of the dendritic tree. In particular, the minimum number of activated spines required to generate an action potential in these cells was at least two spines in the terminal dendrite, three in the trunk and four in the deep dendrites.

### Determination of the origin of the action potentials

**Figure 4:**
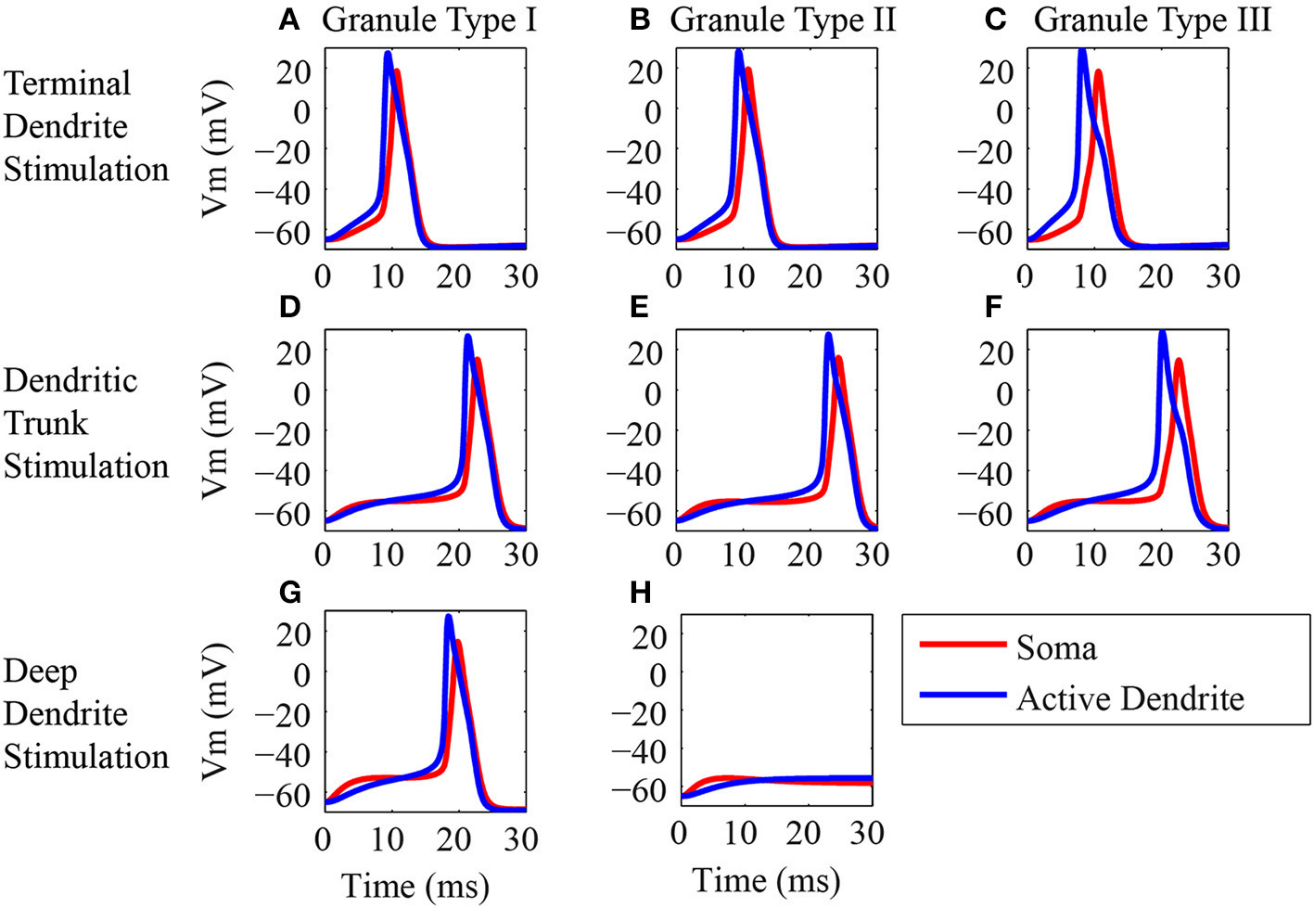
**Origin of the action potentials. (A–H)** Generation of action potentials in the dendritic trunk (blue line) and soma (red line) of the three classes of granule neuron models in response to synaptic activation in different locations of the dendritic tree. The left column **(A,D,G)** shows the responses of the type I granule neuron model, the middle column **(B,E,H)** shows the responses of the type II granule neuron model, and the right column shows the responses of the type III granule neuron model **(C,F)**. The top row shows the responses to stimulations of the spines located in the terminal dendrite **(A–C)**. The middle row **(D–F)** shows the responses to stimulations of the spines located in the dendritic trunk. The bottom row shows the responses to stimulations of the spines located in the deep dendrite. Note that the type III granule neuron model has no spines in the dendrite. The stimulus consists in the synaptic activation of NMDA and AMPA receptors by glutamate in the dendritic spines.

We performed simulations to determine whether the action potentials initiate first in the soma or in the active dendrites in response to synaptic inputs (Figure 4). The stimuli consist of the synaptic activation of NMDA and AMPA receptors on all the spines located in the tip of the terminal dendrites, on the three spines located in the trunk or on all spines located in the tip of the deep dendrites. In each of these three locations the number of activated spines followed the number of dendritic branches of each type of granule cell. The action potentials were recorded simultaneously in soma and in the terminal region of the trunk to determine whether it was generated first in the soma or in the terminal dendrites.

Similar responses were obtained to the stimulation of all the spines in the tip of the terminal dendrites for all classes of granule cells (Figures 4A–C) (Supplementary Figures 2–4). EPSP begins first in the terminal dendrite that reaches the threshold and generates an action potential in the trunk (blue traces) followed by an action potential in the soma (red traces). Equivalent responses occurred to the stimulation of the spines located in the dendritic trunk of all three granule cell types (Figures 4D–F). The action potential was generated first in the trunk (blue trace) followed by an action potential in the soma (red trace).

The stimulation of the spines on the deep dendrites of the type I granule cell (Figure 4G) generated an EPSP that reached a higher value first in the soma (red trace), but the trunk reached the threshold earlier and the action potential was generated first in the dendrite (blue trace) followed by an action potential in the soma (red trace). The type II granule cell did not generate action potentials (Figure 4H), and the EPSP reached a higher value first in the soma (red trace) followed by a higher amplitude in the dendrite (blue trace). Because the type III granule cell model has no spines in the deep dendrites, it was not stimulated in this region.

**Figure 5:**
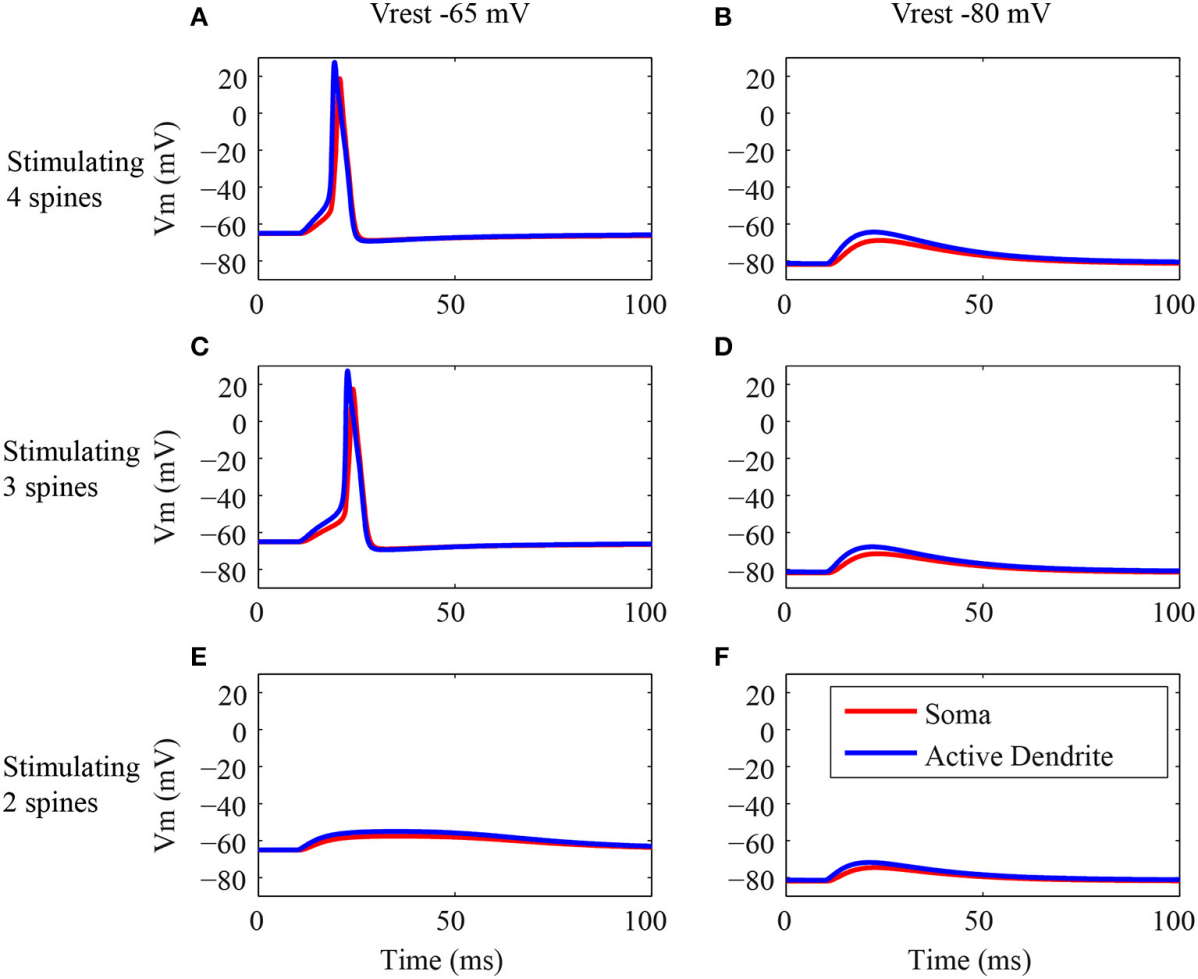
**Electrical responses in the active dendrite and soma. (A–F)** EPSP and action potential generation in the dendritic trunk (blue line) and soma (red line) of the type I granule neuron model in response to synaptic activation of the terminal dendrite. The first row shows the responses of the type I granule neuron model to stimulation of four spines at the tip of the terminal dendrite **(A,B)**. The second row shows the responses of the same cell model to stimulation of three spines **(C,D)**, and the third row shows the responses to stimulation of two spines at the tip of the terminal dendrite **(E,F)**. Note that the left column **(A,C,E)** shows the simulation results for the regular resting membrane potential of −65 mV, and the right column **(B,D,F)** shows the results for the same stimulus pattern, but during the injection of a hyperpolarizing current of −0.1 nA in the soma leading to a resting membrane potential of −80 mV to avoid the generation of action potentials. The stimulus consists in the synaptic activation of NMDA and AMPA receptors by glutamate in the dendritic spines.

The secondary dendrites of the mitral and tufted cells are tangentially oriented in the external plexiform layer, where the granule cell dendrites are located. The radial orientation of the granule cell processes imply that a given mitral/tufted cell may make synapses only to a small number of spines on any given granule cell (Woolf et al., 1991), depending of the location of the contact of the mitral/tufted secondary dendrites on the terminal dendrites of the granule cells. All spines aligned along a transversal line passing through the terminal dendrites of the granule cells were activated to simulate this tangential orientation of the mitral/tufted secondary dendrites on the terminal dendrites of the type I granule cell. Thus, one line crossed the tip of the terminal dendrites, other line crossed the middle of the terminal dendrites, and another the beginning of the terminal dendrites. Because of the dendritic branching, the tip has four branches, the middle has three branches and the beginning has two branches. Therefore, the dendritic tip had four stimulated spines, the middle had three and the beginning had two stimulated spines. The responses of the type I granule cell to these patterns of stimulation are shown in Figure 5.

The action potentials occurred only when at least three spines were activated (Figures 5A,C). Note that both the EPSPs and action potentials were generated first in the trunk (blue trace) and after in the soma (red trace). The latency of the action potential in response to the activation of four spines (Figure 5A) was lower than the latency in response to the activation of three spines (Figure 5C). The activation of two spines (Figure 5E) generated an EPSP stronger in the dendrite (blue trace) in comparison to the soma (red trace).

The same conditions described above were simulated with a hyperpolarizing current injected in soma to avoid the generation of action potentials (Figures 5B,D,F). These simulations confirmed that always the EPSPs had a higher amplitude in the dendrite (blue trace) and lower amplitudes in the soma (red trace). Also, the higher is the number of acivated spines the higher is the amplitude of the depolarization (Figures 5B,D,F).

## Discussion

This computational study characterized the synaptic inputs in the dendritic spines of three classes of granule cells, and verified the impact of the synaptic activation in different regions of these cells in the generation of EPSPs and action potentials. The type I granule cell model was used to study the impact of the mitral/tufted contacts in three different locations of the terminal dendrites of this cell.

The model results predicted that different numbers of spines should be activated in each different dendritic region to induce action potentials in the granule cells. Woolf and colleagues suggested that mitral/tufted cells can connect only to few spines in the granule cell terminal dendrites with an average number estimated near one (Woolf et al., 1991). If this estimate is correct, the present results predict that probably more than one mitral/tufted cell should connect simultaneously to the same granule cell to induce an action potential in this cell. Moreover, because the recurrent axon connections of the mitral/tufted cells in the granule cell occur mainly in the trunk and deep dendrites, the model results predict that the minimum number of recurrent synapses to produce an action should be of at least three in the trunk and four in the deep dendrites. Although the deep dendrites are shorter than the dendritic trunk, deep dendrites are passive compartments, and more spines are required to elicit an action potential. This higher threshold to fire action potentials in response to synaptic inputs in the deep dendrites of the granule cells suggests that they are less sensitive to modulatory inputs of the CFs coming from different brain regions than to synaptic inputs coming from lateral dendrites of the mitral/tufted cells.

All the spikes occurred first in the terminal dendrites rather than in the soma of the three classes of granule cells, even when the stimulation was delivered in the deep dendrites. If this prediction is correct, the recurrent mitral/tufted cell axon connections in the granule cells should induce action potentials first in the terminal dendrites, which could produce more effective reciprocal inhibition in the mitral/tufted cells.

The study of the activation of the spines in different locations of the terminal dendrites suggested that as far from the soma are the mitral/tufted secondary dendrites connections in the granule cell terminal dendrites as stronger are the induced depolarizations in these cells. It probably should occur because the secondary dendrites of the mitral and tufted cells are tangentially oriented in the external plexiform layer, which imply that depending of the location of the contacts of the mitral/tufted secondary dendrites on the granule terminal dendritic branches, different numbers of spines will be activated. The tip of the terminal dendrites has four branches, the middle has three branches, and the beginning has two branches, which imply that mitral/tufted secondary dendrites passing through these regions can connect to four spines, three spines or two spines, respectively.

The small differences in the responses of the three classes of granule neurons could be justified because the type II and type III granule cell models were adaptations of the type I granule cell model (Bhalla and Bower, 1993). The differential distributions of the spines on the dendrites of the three model cells did not induce strong differences in the electrical responses of these cells, and the three model neurons have a very strong tendency to produce spikes first in the active terminal dendrites rather than in the soma.

There are several canonic computational studies and experimental works in literature showing the origin of the generated action potentials in the different regions of the mitral cells (Bischofberger and Jonas, 1997; Chen et al., 1997, 2002; Shen et al., 1999; O'Connor et al., 2012; Migliore et al., 2014). These computational models and experimental evidence have shown that different intensities of the current or synaptic activation of the dendritic tufts of the mitral cells can shift the origin of the action potentials from the terminal dendrites to the soma. However, computational studies utilizing detailed compartmental models of granule cells with spines were performed only in passive models until now (Woolf et al., 1991). The present work is the first to consider the responses of compartmental granule cell models with active properties to synaptic activation in different dendritic locations. Although the models did not present shifts in the origin of the generated action potentials, they predicted that different quantities of spines should be activated in each region of the dendritic tree to induce action potential in the cells, which have important computational implications in the context of the olfactory bulb circuitry.

Electrophysiological evidence demonstrate that mitral and middle tufted cells differ in the decoding manner of odors in the rat olfactory bulb (Nagayama et al., 2004) and have distinct patterns of axonal projection to the olfactory cortex (Haberly and Price, 1977; Scott et al., 1980; Schoenfeld and Macrides, 1984; Nagayama et al., 2010). Because different types of granule cells connect with distinct classes of mitral and tufted cells (Mori et al., 1983), the diverse odor decoding strategies could be at least in part a property of the different types of granule cells that are renewed constantly in the olfactory bulb by the process of adult neurogenesis (Gheusi et al., 2013; Sakamoto et al., 2014). However, because of the similar responses of the three types of granule cell models to several patterns of synaptic activation, the present study favors the hypothesis that the different coding strategies could be both intrinsic properties of the mitral and tufted cells or emergent properties of the olfactory bulb circuitry. More experimental studies and computational models should be developed to test all these possibilities.

Furthermore, recent evidence have shown the expression of transient receptor potential (TRP) channels in mitral and granule cells of the olfactory bulb (Dong et al., 2012; Stroh et al., 2012). The activation of cationic TRP channels in the granule cells is dependent of NMDARs and affects the calcium dynamics required for release of neurotransmitters from the granule cell spines (Egger, 2008; Stroh et al., 2012). In this way, TRP channels that are absent in the present model may play an important role in the reciprocal synapses between mitral and granule cell with active dendrites, and deserve to be investigated in future modeling studies.

### Conflict of interest statement

The authors declare that the research was conducted in the absence of any commercial or financial relationships that could be construed as a potential conflict of interest.
