# Supplementary material for: Electrical responses of three classes of granule cells of the olfactory bulb to synaptic inputs in different dendritic locations
Source: Front Comput Neurosci. 2014 Oct 13;8:128. doi: 10.3389/fncom.2014.00128 (PMC4197772; doi:10.3389/fncom.2014.00128)
Supplement: Supplementary file 1 [file Presentation1.PDF]

Supplementary Material

Electrical Responses of Three Classes of Granule Cells of the Olfactory Bulb to Synaptic Inputs in Different Dendritic Locations

Fábio M Simoes-de-Souza[1,3]*, Gabriela Antunes[1], Antonio C Roque[2]
[1]Laboratory of Neural Systems, Department of Psychology, Faculdade de Filosofia Ciencias e Letras de Ribeirao Preto, Universidade de Sao Paulo, Ribeirao Preto, Brazil
[2] Laboratory of Neural Systems, Department of Physics, Faculdade de Filosofia Ciencias e Letras de Ribeirao Preto, Universidade de Sao Paulo, Ribeirao Preto, Brazil
[3] Center for Mathematics, Computation and Cognition, Federal University of ABC, São Bernardo do Campo, Brazil

*** Correspondence:** Corresponding Author, Center for Mathematics, Computation and Cognition (CMCC), Federal University of ABC (UFABC), Rua Arcturus, 03, Room 250D, Jardim Antares, 09606-0709, São Bernardo do Campo, São Paulo, Brazil.
fabio.souza@ufabc.edu.br

1. Supplementary Figures and Tables

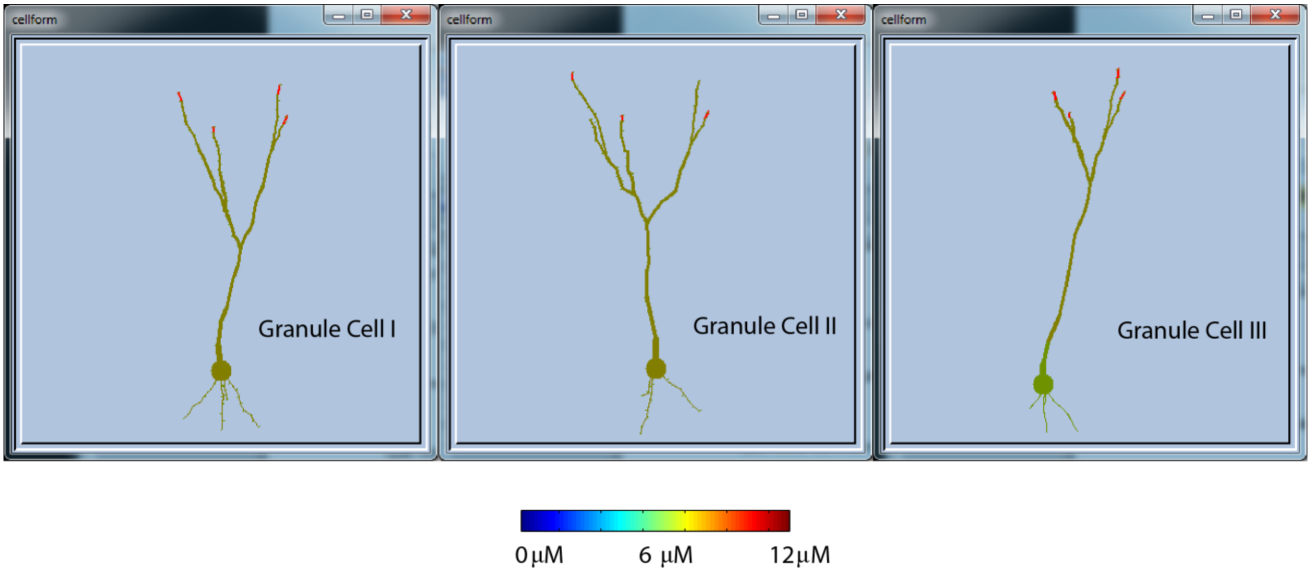

Supplementary Figure 1 Cell-wide visualization of the peak calcium response to synaptic stimulation of AMPARs and NMDARs in the spines located in the tip of the terminal dendrites of three classes of granule cells. The color scale indicates the intracellular Ca^{2+} concentration.

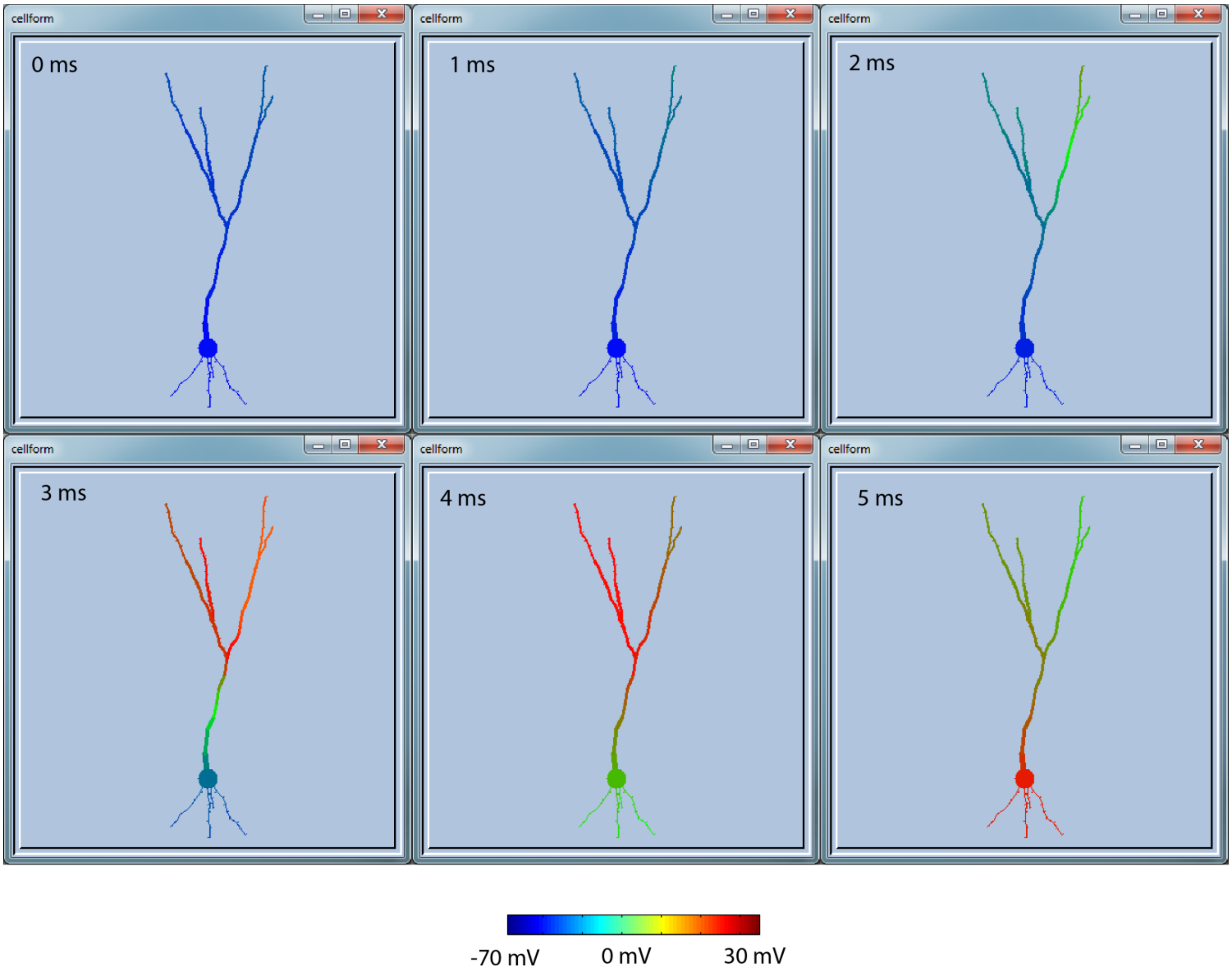

Supplementary Figure 2 Cell-wide visualization of the membrane potential responses to synaptic stimulation of AMPARs and NMDARs in the spines located in the tip of the terminal dendrites of the type I granule cell. The spike initiates first in the terminal dendrites and then propagates to the soma. The color scale indicates the membrane potential.

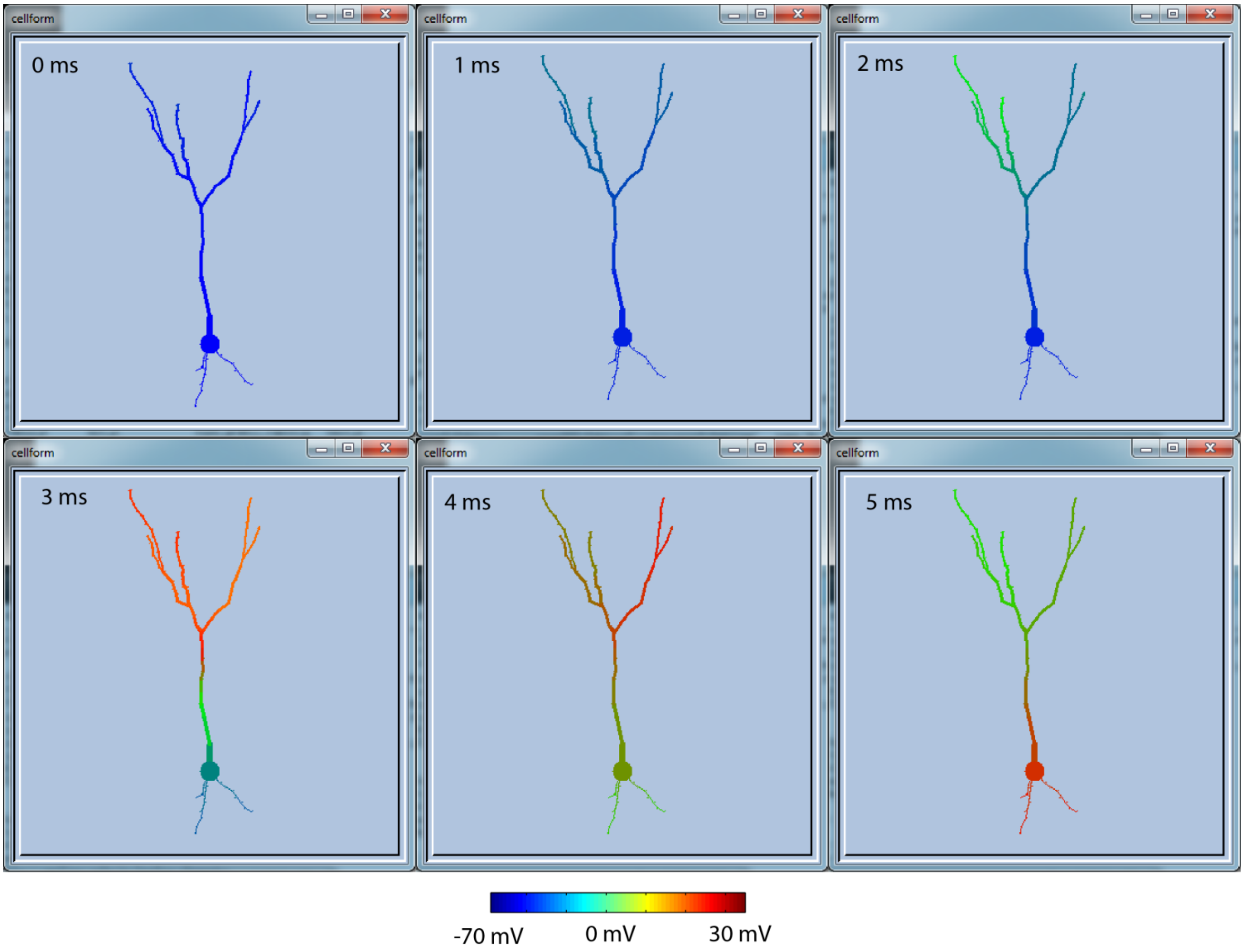

Supplementary Figure 3 Same legend of Supplementary Figure 2 but for the type II granule cell.

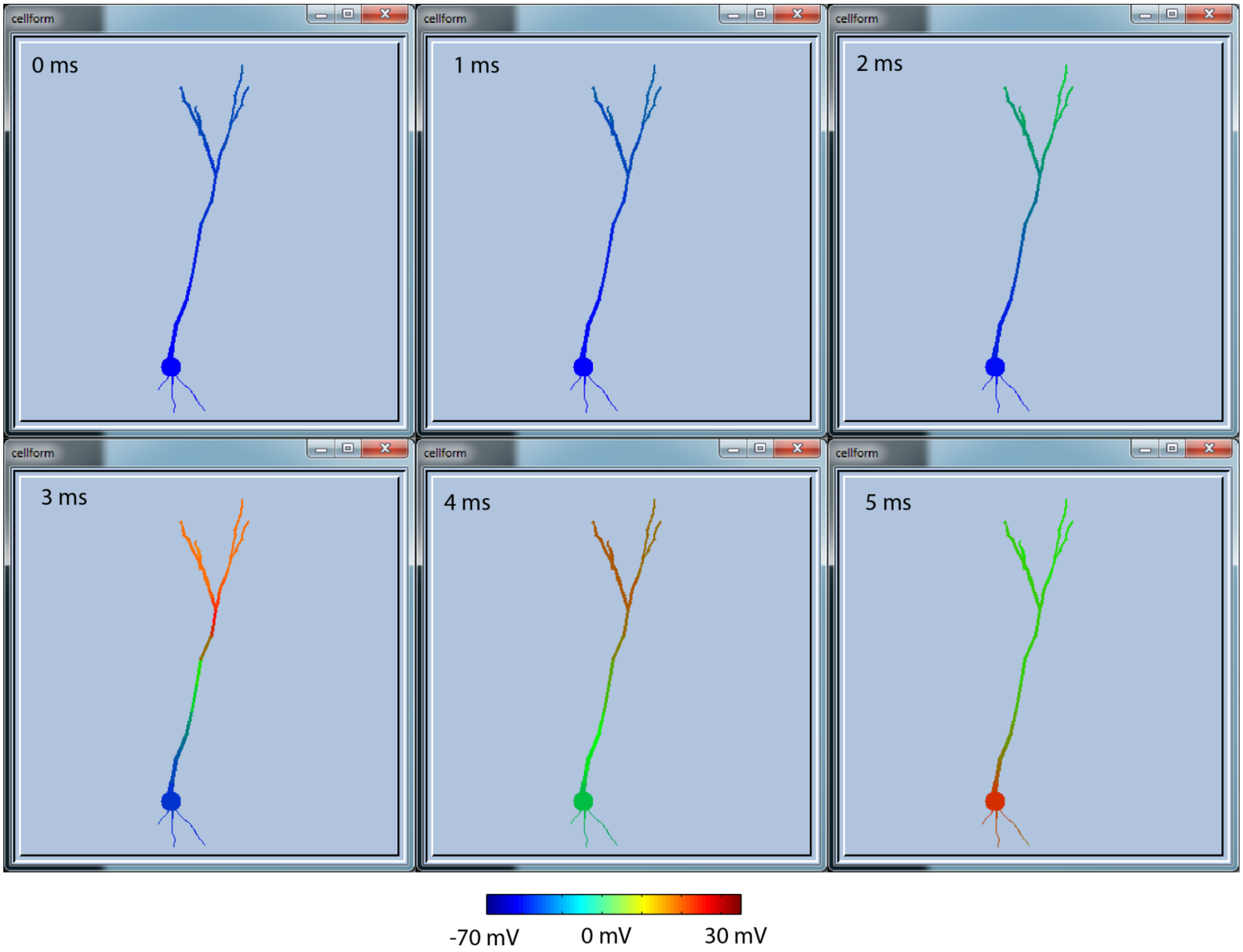

Supplementary Figure 4 Same legend of Supplementary Figure 2 but for the type III granule cell.

Supplementary Table 1 Passive parameters of the three models of granule cell

**Parameter**	**Granule Cell type I**	**Granule Cell type II**	**Granule Cell Type III**
R_a	0.5 KΩcm	0.5 KΩcm	0.5 KΩcm
R_m	120 KΩcm^2	120 KΩcm2	120 KΩcm^2
C_m	1 μF/cm^2	1 μF/cm^2	1 μF/cm^2
Number of Compartments	112	114	89
Number of Spines	112	114	61
V_{rest}	-65 mV	-65 mV	-65 mV

R_a, R_m, C_m, and V_{rest} stand respectively for the specific axial resistance, the specific membrane resistance, the specific membrane capacitance, and the resting membrane potential.
